# Extraction of Alkalis from Silicate Materials PART 2—Crystalline Silicate Materials

**DOI:** 10.3390/ma15176059

**Published:** 2022-09-01

**Authors:** Galyna Kotsay, Wiktor Szewczenko

**Affiliations:** Faculty of Civil Engineering, Mechanics and Petrochemistry, Warsaw University of Technology, Łukasiewicza St. 17, 09-400 Płock, Poland

**Keywords:** cement, extraction, alkalis, strength, mixed alkaline effect

## Abstract

A feature of silicate materials is that they can exist in two antagonistic states—amorphous and crystalline. In both cases, alkalis, which are always present in chemical compositions, play an important role. A feature of alkalis in the composition of silicate materials is that at certain stages of the synthesis of products, they play a positive role, reducing the temperature of synthesis, but worsening the properties of these products at the stage of their operation. Synthesis products should be understood as important building materials such as building glass and cement. It is known that the performance properties of glass and cement can be improved by the extraction of alkalis. In the first part of the article, the issues of extraction of alkalis in amorphous silicate materials-inorganic glasses were considered. This article presents the results of studies on the extraction process of alkalis in crystalline silicate materials-cement.

## 1. Introduction

The influence of alkalis on the clinkerization process and cement properties is ambiguous, affecting the formation of the main clinker minerals and the hydration and modification of products during cement hydration. The performance properties of glass and cement can be improved by the extraction of alkalis. In the first part of the article, the issues of extraction of alkalis in amorphous silicate materials-inorganic glasses were considered [1]. Alkali oxides are introduced into the clinker composition mainly with the help of raw materials used in cement production, mainly from sedimentary rocks, namely clays and marls. Cement raw material mixtures contain an average of 0.5–1.5% alkali [2]. A higher alkali content is typical for the dry method of cement production [3]. The increased alkali content in clinker is also influenced by alternative fuels or waste dust, which contains approximately 5–10% alkali oxides, and their finest particles from electrolytes may exceed 25% [4,5]. In this regard, the production of low-alkaline cement is associated with waste dust.

During the formation of clinker, alkali oxides reduce the temperature of the appearance of the liquid phase to ~1073 K, which contributes to the dissociation of CaCO_3_ and the reaction of CaO with SiO_2_, Al_2_O_3_, and Fe_2_O_3_ [6]. However, at temperatures ~ 1573 K, the alkali affects the acid–base balance of the melt and affects the amphoteric aluminum, and iron ions move into tetrahedral coordination, thereby increasing the viscosity of the melt, which reduces the formation rate of the main clinker mineral, tricalcium silicate (3CaO·Al_2_O_3_), and thus increases the content of undesirable free calcium oxide CaO [4]. In this regard, the allowable alkali content in cement clinker for widely used types of cement does not exceed 1.2%, and for low-alkaline, it is ≤0.6% [7].

In [2,3,8], it is indicated that the apportionment of sodium and potassium in cement clinker components largely depends on the molar ratio of (Na_2_O + K_2_O)/SO_3_. When this ratio is ≤1, sulfates are formed first: Arcanite (K_2_SO_4_), thenardite (Na_2_SO_4_), or double salts: Glaserite (3 K_2_SO_4_ ·Na_2_SO_4_) and langenite (2CaSO_4_ ·K_2_SO_4_). With a ratio (Na_2_O + K_2_O)/SO_3_ ≥1, residual alkalis are bound in silicate, aluminate, or alumino-ferrite phases after forming alkaline sulfates.

In cement mortars, sodium and potassium sulfates or double salts such as glaserite (3 K_2_SO_4_ ·Na_2_SO_4_) and langenite (2CaSO_4_ ·K_2_SO_4_) are soluble in mixing water and quickly diffuse into the cement mortar. However, if sodium and potassium ions are part of the silicate phase (especially belite) or aluminate, they remain in it, slowly decaying for a long time. Table 1 shows the content of various oxides in the resulting clinker mineral phases [6,9].

Compared with other minerals, tricalcium aluminate contains the most considerable impurities. Na_2_O in clinker is mainly bound in the C_3_A phase, where its full content reaches 5.7%. At the same time, K_2_O forms mostly solid solutions in the dicalcium silicate phase. The sodium ion does not cause significant changes in the crystalline phase of clinker minerals because the radius of the sodium ion (0.099 nm) is similar to the ionic radius of calcium (0.106 nm) [10]. However, compared with the sodium ion, the potassium ion has a larger ionic radius (0.133 nm), so when the ion exchange from 2K^+^ to Ca^2+^ it affects the deformation of the phase structure of minerals, which involves its easy dissolution from the clinker. There is 2–3 times more potassium clinker than sodium [9].

Following the Polish-European standard PN-EN 196-2:2006, the content of alkali is limited to their total content. At the same time, according to ASTM C114-11b, the content is evaluated only by soluble alkalis. This method is important when determining a binder′s suitability for autoclaved concrete production [6]. It is believed that the content of alkalis in Portland cement should be below 0.6%; however, literature data indicate that this value may be higher [9,11], depending on the form of their existence in the clinker, as well as on the raw materials and technology used for production.

In some cases, adding alkali to cement can have a positive effect. For example, a small admixture of K_2_S0_4_ added to fast-setting cement intensifies cement hardening during the initial hydration period due to its nucleation properties [2,12]. The acceleration of cement bonding with alkali increases the initial strength of the cement product; however, after a long period of hydration, it reduces the strength. This effect of alkali on cement strength has been confirmed in many scientific studies [4,11,13,14,15,16,17], as shown in Table 2.

At the same time, using alkali in the form of Na_2_CO_3_ and K_2_CO_3_ is undesirable since carbonates react with Ca(OH)_2_, which is formed during the hydration of cement, to give CaCO_3_, which causes the cement to set immediately [3,12,18]. In addition, free alkalis formed during cement hydration can actively interact with reactive silica as part of the filler in concrete, forming products that increase in volume over time, which can lead to its destruction due to alkaline corrosion.

A similar phenomenon of alkaline corrosion is possible, for example, in storing samples of cement products in water. According to the European standard EN 197-1, 27 different types of cement are commonly used today. All of them differ in chemical composition and properties. They have the mechanical strength classes in common, which are determined per the European standard PN-EN 196-3. One of the requirements of this standard is the storage of samples for 28 days in water at a temperature of 20 +/− 1 °C. As is known, the purpose of such treatment of specimens is to accelerate the hydration process and obtain the maximum possible increase in mechanical strength. However, this does not take into account the processes of extraction of alkalis from the cement product, which leads to an increase in the total alkalinity (up to pH = 11–12) of the solution in which the cement product is located. Thus, the storage of cement samples in water, on the one hand, promotes hydration, and on the other hand, the specimens are exposed to an alkaline solution that promotes alkaline corrosion [19], i.e., the strength of cement samples is affected by two mutually exclusive factors. This raises the question of the advisability of storing cement samples in water for 28 days, as provided for in EN 196-1. This issue is addressed in the studies, which are presented below.

The main reason for the increase in pH is the diffusion of calcium hydroxide into the solution as a product of hydration; however, the alkaline cations of sodium and potassium, which are part of the cement, also contribute to this process. For low-alkaline cement, this contribution is small; however, if glass powders or water glass with high alkaline activity are introduced into the cement composition, this contribution can be very significant [20]. In connection with the preceding, we attempt to investigate the effect of various alkali-containing additives on the process of extraction of alkalis from cement samples.

## 2. Materials and Methods

Portland cement CEM I 42.5N produced by Cement Ożarów S.A. (Ożarów, Poland) [21] was used as the main object of research. As alkali-containing additives, sodium and potassium water glass (WG) produced by [22] were used. The chemical compositions of the materials are shown in Table 3.

The flexural and compressive strength tests of the cement mortars were performed according to [23]. The specimens were prepared from CEM I 42.5N (450 g), standard sand (1350 g), and distilled water (225 g). The tests were carried out on specimens of mortar sized 40 mm × 40 mm × 160 mm after 2 and 28 days of forming. 

To determine the alkaline activity of cement products, distilled water was used as an extractant with a ratio of the specimen surface to the extractant volume of 0.34 cm^−1^. The specimens of mortars were made in molds sized 40 mm × 40 mm × 160 mm. All cuboids of mortars were taken out from the molds after one day, and the alkaline activity of the mortar was then determined. For the quantitative analysis of alkali content, a flame photometer FP902 (PG Instruments Limited, Alma Park, Wibtoft Leicestershire, UK) with an accuracy of +/−0.5% was used. The results of the alkaline activity of specimens are presented in units of ppm/m^2^ of mortar. Every property of mortar analyzed was evaluated on a series of six specimens.

## 3. Results

The action of alkali is ambiguous; on the one hand, they accelerate the setting and hardening of cement, but on the other hand, they affect the corrosion of concrete. The content of easily soluble salts is of great importance in the extraction of alkali in cement mortars. However, it was previously established that the introduction of glass waste or sodium water glass into the cement composition leads to blockage of the extraction process of potassium cations. This process is called the mixed alkali effect, by analogy with glass.

**Figure 1:**
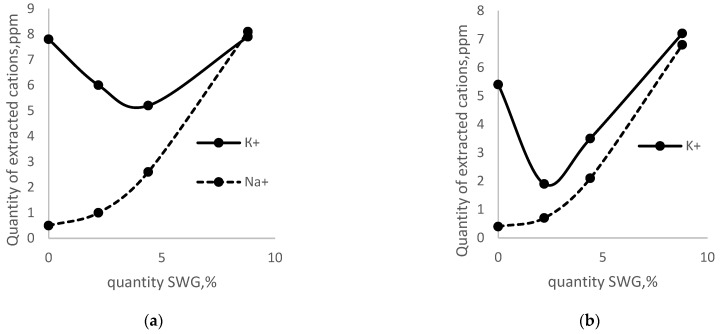
The amount of extracted cations depends on the amount of addition of sodium water glass: (**a**) after 24 h, (**b**)—after 48 h.

Figure 1 shows the amount of extracted alkali cations with the addition of sodium water glass (SWG). First of all, the decrease in the amount of extracted potassium cations with the addition of sodium WG should be noted, in an amount of 0.5 to 5% after 24 h of cement hydration and from 0.5 to 2.5% after 48 h of hydration of CEM I 42.5N. Previously, a similar effect of suppressing the alkaline activity of K+ cations (mixed alkaline effect) was described in [19,22], using the example of fast-setting cement CEM I 32.5R.

In the glass, the mixed alkaline effect is explained by an increase in the binding forces between alkali cations and oxygen, which leads to a change in the degree of polarization of non-bridging oxygen ions and, as a result, a decrease in the mobility of cations with a smaller ionic radius (r (Na^+^) = 0.95 nm) by cations with a large ionic radius (r(K^+^) = 1.33 nm) [24,25].

**Figure 2:**
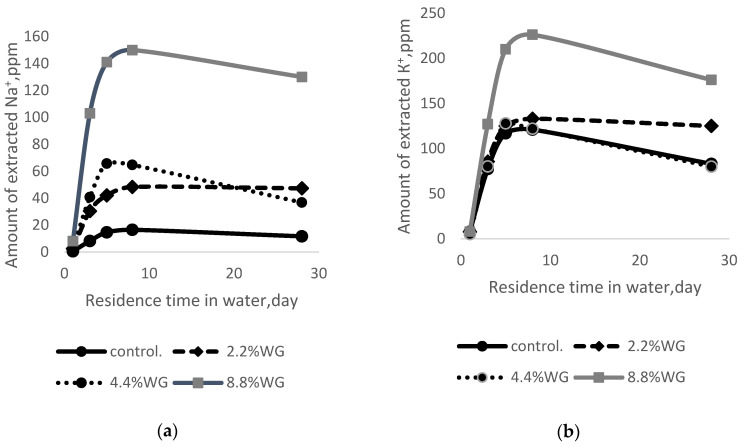
Dependence of the amount of extracted alkali cations on the amount of addition of sodium WG and residence time in the water: (**a**) Na^+^; (**b**) K^+^.

In cement, the mixed alkaline effect (see Figure 2) can be explained by the formation of SiO_2_ gel, which adheres to the pore walls, reducing their diameter, which leads to blocking large potassium cations and blocking their diffusion into the extractant solution.

Extraction of the alkaline ingredients of the cement occurs during the standard storage of cement samples in water. After 24 h of molding, the beams were placed in a container with extractant-distilled water. After the specimens were immersed in water, after 30 s, a sample of the extractant was taken in an amount of 30 mL, which was then analyzed on a flame photometer for the content of sodium and potassium cations. In the same way, the samples were tested after 3, 5, 8, and 28 days.

**Figure 3:**
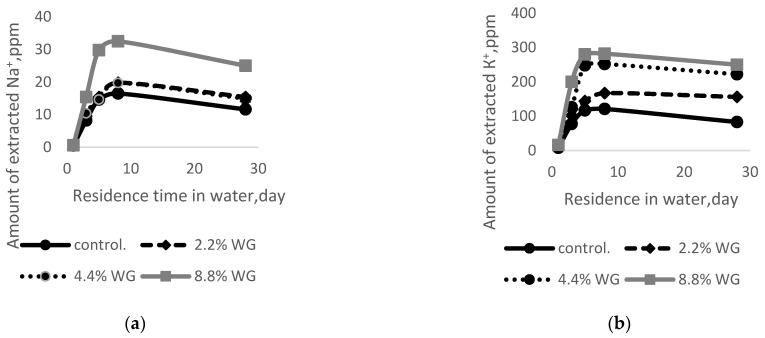
Dependence of the amount of extracted alkali cations on the addition of potassium WG and time in the water: (**a**) Na^+^; (**b**) K^+^.

During the active extraction process, the amount of extracted sodium cations increases with an increase in added WG (see Figure 3a). For example, after five days, the amount of Na+ extracted from cement samples containing 2.2% sodium WG is 2.8 times higher than in the control sample without additives, while for 4.4% and 8.8%, it is 4. 5 and 10 times higher. The amount of extracted K^+^ for the control sample and the sample containing 2.2% and 4.4% SWG is approximately the same level, and a sharp increase is observed only by 8.8%.

These results indicate potassium cations are blocked by adding SWG up to 4.4%, and the mixed alkaline effect disappears. A similar picture is observed on the curves of the amount of extracted alkali cations on the amount of addition of potassium water glass and the residence time in water (Figure 3). However, in this case, adding up to 4.4% potassium WG blocks the extraction of sodium cations (Figure 3a), while potassium cations freely diffuse into the surrounding solution. Thus, regardless of the type of WG additive, its introduction from 2.2% to 4.4% blocks the extraction of the second alkaline cation, which once again confirms the presence of the mixed alkaline effect.

Figure 2 and Figure 3 show sodium and potassium cations diffuse from the cement into the surrounding water. At the same time, their concentration increases within 5–8 days and then decreases. The diffusion process is most active in the first five days and equalizes the concentration of alkaline cations in the cement and extractant. In fact, at ordinary temperatures, diffusion is only due to differences in the number of alkali cations. The decrease in the concentration of alkaline cations after eight days can be explained by the formation of new products in the composition of the extractant.

Comparing the results presented in Figure 2 and Figure 3, it should be noted that, in addition to a large difference in the amount of extracted cations, the pattern of changes in extraction over time is almost the same for sodium and potassium cations. In addition, when adding potassium water glass, there is no mixed alkaline effect, which is observed when adding sodium water glass.

When a cement sample is kept in water (at 20 °C), the latter is saturated with Ca^2+^, K^+^, Na^+^, and OH^-^ ions, which leads to the formation of alkali metal hydroxides and an increase in the pH of the extractant to 12–13. The penetration of the latter into the porous surface layers of cement samples leads to alkaline corrosion. To determine the possible effect of alkaline corrosion on the mechanical strength of the samples, mechanical strength tests were carried out after two days of storage in air (early strength), as well as after 28 days of storage of samples in a closed container and water, by EN 196-1 (Table 4).

First of all, it should be noted that the cement CEM I 42.5N used in the studies complies with the European standard EN 197-1 both in terms of early strength (after two days) and after 28 days of storage. With an increase in the amount of water glass, the strength decreases by 50% or more regardless of its type. This may be due to an increase in the porosity of the samples, as evidenced by a decrease in density.

For the control sample (without additives), the compressive and tensile flexural strength after 28 days of storage is almost the same for specimens stored in air and water. When adding 2.2% to 4.4% WG, the strength also practically does not change (it changes within the error), and only when 8.8% sodium and potassium WG are added is an almost twofold decrease in strength observed after the storage of samples in water. Taking into account the fact that when adding 8.8%, the amount of extracted alkalis increases sharply (see Figure 3a,b), the concentration of alkali hydroxides in the extractant composition increases in parallel, which leads to an increase in alkaline corrosion and a decrease in the strength of the samples.

## 4. Conclusions

Studies of alkaline extraction in antagonistic silicate materials have shown that, regardless of the state of the silicate material, the alkaline component is the least bound in its structure. Possessing low activation energies, alkali cations easily leave their places and, due to the difference in concentration with the extractant, pass into the surrounding solution, forming the corresponding hydroxides. The newly formed alkaline environment is chemically aggressive and penetrates into the pores of the cement stone, leading to external and internal corrosion of the material. Short-term exposure to the water makes it possible to extract the alkaline cations from the glass surface, which characterize its alkaline activity, and this can be corrected by treating the glass powder with hot water. The addition of sodium and potassium water glass into the mixing water in an amount of 2.2–4.4% makes it possible to partially block the process of extracting alkali cations from Portland cement due to the mixed alkaline effect.

## Figures and Tables

**Table 1:** Content of admixtures in clinker mineral phases (wt.%).

Phase (Shortened Cement Chemical Notion ^1^)	Mineralogical Term	Contents of Admixtures [wt.%]						
		Al_2_O_3_	Fe_2_O_3_	MgO	SiO_2_	Na_2_O	K_2_O	TiO_2_
Tricalcium silicate (C_3_S)	Alite	0.7–1.7	0.4–1.6	0.3–1.0	-	0.1–0.3	0.1–0.3	0.1–0.4
Dicalcium silicate (C_2_S)	Belite	1.1–2.6	0.7–2.2	0.2–0.6	-	0.2–1.0	0.3–1.0	0.1–0.3
Tricalcium aluminate (C_3_A)	Aluminate	-	4.4–6.0	0.4–1.0	2.1–4.2	0.3–1.7	0.4–1.1	0.1–0.6
Tetracalcium aluminoferrite (C_4_AF)	Brownmillerite	-	-	0.4–3.8	1.2–6.0	0.0–0.5	0.0–0.1	0.9–2.6

^1^ In this paper was used abbreviations according to Standard cement chemistry: C = CaO; S = SiO_2_; A = Al_2_O_3_; F = Fe_2_O_3_).

**Table 2:** Influence of alkalis on the strength of Portland cement [4].

Hydration Time	Compressive Strength [MPa]	
	Content in Cement 1 0.6% Na_2_O+K_2_O	Content in Cement 2 1.6% Na_2_O+K_2_O
12 h	5.9	6.3
1 day	21.7	22.9
3 days	52.8	54.2
7 days	67.1	67.1
14 days	83.8	83.2
28 days	85.7	84.8

**Table 3:** Chemical compositions of materials.

Materials	Oxides (wt%)								
	SiO_2_	Al_2_O_3_	Fe_2_O_3_	CaO	MgO	Na_2_O	K_2_O	SO_3_	H_2_O
CEM I 42.5N	21.26	4.13	5.40	64.21	1.88	0.13	0.47	2.52	-
Sodium water glass (SWG)	26.14	-	-	-	-	7.86	-	-	66.00
Potassium water glass (PWG)	23.56	-	-	-	-	-	6.58	-	69.86

**Table 4:** Dependence of strength on the exposure time and storage conditions of CEM I 42.5N beams.

The Composition	Two Days in Air		28 Days in Air		28 Days in Water		Density, g/cm^3^
	R*, MPa	R**, MPa	R*, MPa	R**, MPa	R*, MPa	R**, MPa	
CEM I 42.5N-100%	5.4	23.1	8.9	46.5	9.0	47.5	2.306
CEM I 42.5N +SWG-2.2%	5.3	20.7	8.2	38.3	8.7	36.7	2.316
CEM I 42.5N +SWG-4.4%	5.1	17.0	7.2	31.4	7.8	30.9	2.256
CEM I 42.5N +SWG-8.8%	3.2	11.5	4.6	24.2	0.8	12.5	1.723
CEM I 42.5N +PWG-2.2%	5.5	24.5	8.6	50.0	8.5	52.0	2.267
CEM I 42.5N +PWG-4.4%	5.2	21.4	8.2	40.2	8.1	41.0	2.278
CEM I 42.5N +PWG-8.8%	1.5	15.8	1.7	14.6	0.7	8.3	1.949

R* bending tensile strength; R** compressive strength.

## Data Availability

Data are available in a publicly accessible repository.
